# Understanding Sleep Challenges in Pediatric Palliative Care: A Patient-Focused Scoping Review and Implications for Practice

**DOI:** 10.3390/jcm15020438

**Published:** 2026-01-06

**Authors:** Giorgia Varallo, Anna Mercante, Valentina Baldini, Martina Gnazzo, Caterina Testoni, Franca Benini, Sergio Amarri, Maristella Scorza, Sandro Rubichi, Fabio Pizza, Giuseppe Plazzi

**Affiliations:** 1 Department of Biomedical, Metabolic, and Neural Sciences, University of Modena and Reggio Emilia, 41125 Modena, Italy; valentina.baldini@unimore.it (V.B.); martina.gnazzo@unimore.it (M.G.); maristella.scorza@unimore.it (M.S.);; 2 Department of Biomedical and Neuromotor Sciences, University of Bologna, 40125 Bologna, Italy; mercanteanna@outlook.com (A.M.);; 3 IRCCS Istituto delle Scienze Neurologiche di Bologna, 40139 Bologna, Italy; 4 Pediatric Palliative Care, Pain Service, Department of Women’s and Children’s Health, University of Padua, 35128 Padua, Italy; franca.benini@aopd.veneto.it; 5 Fondazione Hospice Maria Teresa Chiantore Seragnoli, 40139 Bologna, Italy; sergio.amarri@fondazionehospiceseragnoli.org

**Keywords:** pediatric palliative care, sleep, life-limiting conditions, life-threatening conditions, sleep disturbances

## Abstract

Sleep disturbances are prevalent among children receiving pediatric palliative care (PPC) and have a substantial negative impact on the quality of life of both patients and their families. This scoping review aims to examine further key factors contributing to sleep disturbances in PPC by analyzing available clinical studies, focusing on child-related aspects such as pain, repositioning, and epilepsy, as well as environmental factors such as light, noise, and the care setting. In addition, we discuss the critical role of interdisciplinary collaboration in improving sleep outcomes for this population and provide clinical implications. Future research should prioritize developing evidence-based interventions to optimize sleep and enhance the well-being of children with PPC needs and their caregivers.

## 1. Introduction

Sleep is a fundamental and multifaceted physiological process that plays a key role in maintaining optimal health and well-being, especially during childhood. It significantly affects early-life development [1,2], encompassing several brain functions, including optimal growth [3,4], development [5], synaptic homeostasis, as well as restorative functions, energy conservation, and immune system regulation [6,7,8]. Sleep also profoundly influences physical, emotional, and cognitive health, with insufficient or poor sleep quality associated with impaired attention, memory, and learning difficulties that may negatively affect academic performance and cognitive functioning [9]. Additional findings indicate that inadequate sleep may increase irritability, anxiety, and depression symptoms [9,10], whereas adequate sleep has been associated with improvements in emotional regulation, empathy, social competence, and behavior [11]. In the context of patient care, research has demonstrated that sleep disturbances have a greater occurrence in acute and chronic medical conditions [12] and are linked to the worsening of various symptoms, including pain [13], inflammation [14], and cognitive decline [15], ultimately leading to reduced quality of life (QoL) and increased healthcare costs [16].

In pediatric palliative care (PPC), sleep disturbances have been recently recognized as one of the most challenging symptoms, yet the evidence remains limited [17].

The World Health Organization (WHO) defines PPC as comprehensive, active care provided to pediatric patients with complex life-limiting (LLCs) or life-threatening (LTCs) conditions and their families. Its goal is to achieve the best possible well-being and QoL by preventing and relieving suffering and addressing the evolving physical, psychosocial, and spiritual needs of patients and their caregivers through a shared and interdisciplinary team approach [18,19]. Life-limiting conditions typically refer to chronic diseases or syndromes that, while not necessarily immediately life-threatening, have no prospect of cure and carry the likelihood of significantly reducing the child’s lifespan, or where the primary need for palliative care is not an expected abbreviation of life but rather the quality of that life [20]. Life-threatening conditions include those that are active, progressing, or advanced, where curative treatment is possible but can be unsuccessful or present immediate threats to the child’s life, leading to a significant risk of incurability and increasing the complexity of care [21,22]. The clinical spectrum of PPC is, therefore, broad and heterogeneous, including oncological and non-oncological medical disorders, such as neurological, metabolic, cardiological, and respiratory disorders due to congenital, perinatal, or acquired conditions [23]. Currently, the estimated number of children eligible for PPC worldwide exceeds 20 million and is expected to rise in the coming decade [24,25,26].

In this context, disordered sleep contributes to additional burden and health concerns, warranting special attention given their recurrence and far-reaching impact on multiple aspects of children’s and families’ functioning and QoL [27,28]. These disturbances also constitute a major concern for parents, representing one of the most common symptoms requiring intervention [29]. Their phenomenology is multifactorial and influenced by various factors, necessitating a multidimensional approach to optimizing care. Despite their importance as primary or co-morbid conditions [30,31,32,33,34,35,36], sleep disturbances are not routinely addressed and often remain underdiagnosed and undertreated [37].

With this scoping review, we aim to examine key, real-world contributing factors to sleep disturbances in PPC through a focused, patient-based analysis of available clinical studies. Secondly, the review emphasizes implications for practice and knowledge gaps in the assessment and management of sleep problems in this population and highlights their clinical relevance. The overarching aim is to delineate priority areas for future research and to support the development of more tailored, evidence-informed approaches to sleep care in this particular setting.

## 2. Materials and Methods

Firstly, a literature search was conducted that included PubMed, Medline, and Embase databases using the following study terms: ‘pediatric palliative care’ AND ‘sleep disorders’ or ‘sleep disturbances’ or ‘sleep’. Only English and Italian-language published studies in peer-reviewed journals were included. Studies conducted since 1 January 2000, were included to reflect contemporary critical care practice. Studies were included if they (i) focused on pediatric patients (age range: 0–18 years), (ii) considered life-limiting or life-threatening conditions with PPC needs, (iii) evaluated sleep disturbances through a clinical diagnosis or a parent or self-report questionnaire, (iv) examined child-related or environmental-related factors contributing to sleep disturbances, and (v) had a cross-sectional or prospective design. Studies were excluded when they did not meet the criteria or were not available in full-text format. Additionally, review and opinion articles were excluded, but their reference lists were screened for relevant primary studies. Due to the scoping nature of this review, no formal data synthesis was conducted. The findings were qualitatively analyzed and presented. Given the limited number of PPC-specific findings on this topic, related pediatric literature was selectively drawn upon and referenced when necessary to support interpretation and facilitate contextual discussion, without implying direct comparison.

## 3. Results

Only three studies [30,38,39] met all inclusion criteria for evaluating factors associated with or contributing to sleep disturbances in the PPC population. Table 1 summarizes the main characteristics and findings of each study.

Dreier et al. [30] conducted a cross-sectional observational study in Germany examining sleep problems in severe psychomotor impairment (SPMI) and the influence of child-related and environment-related factors on sleep quality. The study analyzed 212 children (mean age 10.4 years) recruited from three inpatient institutions (children’s hospital, hospice, respite care) and one outpatient pediatric palliative home care service. The diagnostic profile reflected the complexity of PPC populations: the most common conditions were cerebral palsy (26.3%), global developmental delay of unknown origin (17.1%), rare syndromes (13.7%), and metabolic or neurodegenerative disorders (10.2%). More than half of the sample had comorbid epilepsy (55.8%), and 84.4% received medication, including anticonvulsants (61.6%) and analgesics (9.6%). Sleep disturbances were assessed using the Sleep Questionnaire for Children with Severe Psychomotor Impairment (SNAKE), a validated caregiver-report measure addressing sleep quality, sleep duration, sleep efficacy, and five sleep problem domains. Predictors of sleep disruption included pain, nighttime repositioning, epileptic seizures, and medical care, as well as environmental factors such as light, noise, TV/radio, and open doors in the sleeping environment.

Marinetto et al. [39] conducted a cross-sectional observational study to evaluate sleep quality and its association with caregiver burden among children receiving care at a regional PPC center in Italy. The study was carried out between August and September 2020 and included 142 children and adolescents after exclusion due to language, refusal, or late enrollment. The sample was predominantly non-oncological (87%), encompassing complex conditions such as neuromuscular, metabolic, genetic, and neurological disorders, with only 6% affected by oncologic diseases. Sleep was assessed through a parent-reported 9-item questionnaire, developed based on existing pediatric sleep literature and partly adapted from the SNAKE instrument. Data on nocturnal awakenings, pharmacological sleep support, environmental factors, and caregiver-reported sleep quality were collected alongside demographic and clinical information, including medical device use and physiotherapy needs. Key features of the care context included the frequent use of medical devices (76%), home- and hospice-based PPC support, and substantial overnight care demands.

Harputluoğlu et al. [38] conducted a single-center, cross-sectional study in Türkiye (2020–2021) investigating sleep disorders among children receiving PPC and their primary caregivers. The study included 76 parent–child dyads aged 1 month to 18 years, the majority being male children (61.8%) and female caregivers (94.7%). Clinical conditions were predominantly neurological, with 52.6% diagnosed with cerebral palsy and 84.2% bedridden, reflecting a population with highly complex healthcare needs. Sleep disturbances were evaluated using the Children’s Sleep Habits Questionnaire (CSHQ) for children and the Pittsburgh Sleep Quality Index (PSQI) for caregivers. Nearly all children (98.7%) met the clinical cutoff for pediatric sleep disorder, and 88.2% of caregivers reported poor sleep quality. The study further analyzed demographic and clinical correlates, demonstrating a weak but significant positive correlation between child sleep disturbances and caregiver sleep quality.

## 4. Discussion

The few studies yielded by our review underscore the limited evidence on factors contributing to sleep disturbances in children requiring PPC. However, they provide preliminary insights into real-world contributors that raise several points of interest for further discussion.

### 4.1. Child- and Disease-Related Factors

#### 4.1.1. Pain

A strong association between pain and sleep disturbances was identified in the study conducted by Dreier et al. [30]. Their findings showed that children who had pain in the past four weeks were over four times more likely to have poor sleep. Indeed, pain emerged as the most influential factor in predicting sleep difficulties, including trouble falling asleep, staying asleep, arousal and breathing problems, and daily behavioral problems. On the contrary, no significant association was observed between pain and daytime sleepiness. Similarly, in the sample analyzed by Marinetto et al., pain or crying triggered nocturnal awakenings in up to 14% of cases, with nearly one-third of patients experiencing frequent nighttime sleep disruptions [39].

The link between sleep disturbances and pain is well established, and a positive correlation has previously been demonstrated across various pediatric conditions outside PPC, namely, intellectual and developmental disabilities [40,41,42], acute trauma, headache, juvenile idiopathic arthritis, and other chronic disorders [43,44]. In addition to acting as a direct noxious stimulus, pain stimulates the sympathetic nervous system [45] and activates the hypothalamic–pituitary–adrenal–thyroid–gonadal system [46], which is the primary mechanism that regulates the stress response, possibly further contributing to arousals that cause sleep disturbances [39]. Notably, pain is often associated with symptoms of anxiety and depression, which are also known to possibly alter the sleep–wake cycle [47]. Thus, the added burden of pain can further exacerbate psychological distress and, in turn, aggravate sleep disturbances, creating a vicious cycle.

In the context of PPC, identifying pain may be particularly challenging, especially in non-verbal patients [48]. Caregivers and clinicians must actively observe and interpret pain-related behaviors such as vocalizations of distress (e.g., moaning or crying), facial expressions such as grimacing, signs of agitation/irritability, changes in behavior or eating/sleeping patterns, as well as variations in autonomic parameters [49].

#### 4.1.2. Epilepsy

Concomitant epilepsy may represent an additional risk factor for the development of sleep disturbances. The only study examining this issue found that epilepsy constituted the strongest predictor of daytime sleepiness and was associated with significantly lower sleep efficiency in children with life-limiting conditions and SPMI [30]. Children are likely to be awakened multiple times as a result of nocturnal seizures, resulting in possibly increased daytime fatigue. It is not clear whether the association between epilepsy and sleep disturbances is limited to nocturnal arousals caused by seizures or whether it includes endogenous defects in sleep regulation [30,50,51], such as abnormal melatonin secretion [52]. Growing evidence suggests bidirectional interactions among sleep, circadian rhythm, and epilepsy [53,54]. More importantly, the use of anticonvulsants can negatively affect sleep onset and efficiency, and increase daytime somnolence and arousal [51,52,55].

#### 4.1.3. Clinical and Supporting Care Activities

##### Repositioning

Prolonged or forced bedboundness—resulting from medical procedures or limited mobility due to the underlying condition or its complications—often causes physical discomfort and necessitates postural repositioning both at night and during the day. In the study by Dreier et al., nighttime repositioning was associated with a three-fold increased likelihood of poor overall night sleep quality, making it the second most significant factor affecting sleep [30]. Repositioning also emerged as a significant predictor of both sleep duration and sleep efficacy and was further associated with sleep difficulties in falling asleep, arousal and respiratory issues, and daytime sleepiness. Consistently, Marinetto et al. reported that nocturnal awakenings were primarily triggered by the need to change position. The impact of nocturnal repositioning on sleep has been previously highlighted in male children with Duchenne muscular dystrophy, where repositioning was associated with a seven-fold increased likelihood of developing sleep disturbances [56].

##### Other Needs for Nocturnal Care and the Use of Medical Devices

Beyond repositioning, nighttime care did not show a significant impact on sleep parameters in children with life-limiting conditions and SPMI [30] in the study conducted by Dreier. In line with this, Marinetto also found no significant correlation between the use of medical devices and sleep quality [39]. A plausible explanation for these findings lies in the broad, non-specific nature of the term “medical care,” which encompasses a wide range of interventions and practices that may variably affect sleep, including medication administration. Additionally, many patients with PPC needs are dependent on technological supports, the most common being nutritional and respiratory devices. Noise generated by nutritional and suction pumps, mechanical ventilation (invasive or non-invasive), and alarm systems, including those from ostensibly non-invasive instruments such as pulse oximeters, may plausibly interfere with sleep (see below).

### 4.2. Environmental-Related Factors

#### 4.2.1. Light

Evidence on the influence of light on sleep in the PPC setting was scarce. In the study by Dreier et al., light exposure was not a significant predictor of sleep disturbances [30]. This result contrasts with some of the existing literature. For example, excessive light exposure was associated with shorter sleep duration and increased nighttime awakenings in a systematic review on hospitalized pediatric cancer patients from different contexts [57]. Moreover, light is recognized as a primary environmental factor that substantially influences the sleep/wake cycle. It plays a crucial role in modulating melatonin levels, in the orientation of circadian rhythm, and in promoting sleep, as well as in cortisol production, related to stress response and alertness. These hormones are closely linked to cognitive functioning, alertness, and sleepiness [58]. Adequate exposure to light of sufficient intensity could reset the circadian pacemaker in humans, leading to decreased melatonin and increased cortisol levels, thereby altering sleep and wake patterns [59].

#### 4.2.2. Noise

Several reports on the adverse effects of nocturnal noise exposure on sleep are available in the general literature. When severe and frequent enough, noise can induce arousal, leading to significant sleep fragmentation and deprivation at relatively low exposure levels and independently of the noise source and environment [60].

In PPC, noise was not found to be associated with sleep quality rating, sleep efficacy, or sleep duration [30].

However, evidence also indicates that noise can affect sleep quality in hospitalized pediatric patients. More specifically, in a study of pediatric oncology patients receiving chemotherapy, nightly ambient noise frequently exceeded WHO-recommended levels, averaging over 45 dB with occasional spikes up to 80 dB, comparable to moderate conversation or sudden loud sounds [61]. The differences in outcomes can be attributed to the different settings and methods used: the first used parental reports, while the other used recordings from dedicated sensors and an actigraph.

#### 4.2.3. Pharmacological Interventions

Only Marinetto et al. examined the association between sleep quality and the use of sleep medications, reporting a significant correlation indicating a trend toward better sleep quality among untreated children [39].

### 4.3. Parent-Related Characteristics and Sleep Quality

Harputluoğlu et al. [38] identified significant differences in children’s sleep quality based on parental characteristics: children of university-educated parents demonstrated lower sleep-related anxiety, parasomnia symptoms were more prevalent among children of illiterate parents, and sleep onset delay was greater in children whose parents were employed. Moreover, parental sleep quality was significantly correlated with the child’s sleep quality, underscoring the potential bidirectional interplay between child sleep disturbances and caregiver well-being. This pattern is consistent with the observations of Marinetto et al., who described a significant correlation between the children’s and caregivers’ sleep quality [39].

### 4.4. Research Gaps

The concerning lack of literature on factors contributing to sleep disturbances in PPC calls for further investigation into several potentially important contributors that remain unaddressed. The following section summarizes factors warranting future research.

#### 4.4.1. Psychological Distress and Other Comorbidities

The first international consensus on sleep problems in PPC recently emphasized that assessing the child’s psychological profile is crucial for understanding and managing sleep disturbances in this population. The panelists largely agreed on the bi-directional relationship between poor-quality sleep and more intense feelings of depression and hopelessness, as well as on the association between deprived sleep and higher rates of anxiety in both patients and their caregivers [37].

Although specific data on sleep and psychopathological disorders in PPC are still lacking, evidence from other pediatric populations widely supports the potential significant role of psychological factors in altering sleep [62,63,64]. Furthermore, anxiety in children has been associated with difficulty falling asleep, nightmares, reduced slow-wave sleep, more frequent awakenings, and shorter overall sleep duration [65]. Similarly, adolescents with mood disorders, such as major depressive episodes, often experience insomnia, prolonged sleep latency, and reduced total sleep time [66,67].

Other frequent comorbidities in these children, such as dystonia and neuroirritability, can substantially disrupt sleep by causing involuntary movements, muscle stiffness, or heightened nighttime arousal. These symptoms often result in frequent awakenings and fragmented sleep, further increasing caregiver burden and complicating the management of sleep disturbances [68,69].

#### 4.4.2. Adverse Effects of Medications

Many medications prescribed for underlying conditions or comorbidities—such as corticosteroids, anticonvulsants, or other essential therapies—can adversely affect sleep, causing insomnia, fragmented sleep, or daytime sleepiness. These drugs are often unavoidable due to their critical role in managing pain, seizures, or other symptoms. Evidence quantifying their impact on sleep, particularly in PPC, remains limited, highlighting another potential research gap in understanding how to optimize sleep while maintaining necessary medical treatment.

#### 4.4.3. Daytime Sunlight Underexposure

Hospitalized patients often experience insufficient exposure to daylight, which can also affect their sleep–wake cycle [70]. In the context of PPC, patients often have movement impairments or activity limitations due to underlying conditions or need to remain bedridden for disease progression or clinical complications, further restricting daytime sunlight exposure, which may constitute another potential element influencing the sleep–wake cycle.

#### 4.4.4. Use of Electronic Devices

The lives of children and adolescents are deeply influenced by the widespread use of interactive electronic devices such as smartphones, tablets, and portable gaming devices, as well as social networks, with their increased usage at bedtime linked to a decline in the quality of their sleep [71,72]. In addition to data from the general pediatric population, some evidence has been replicated in pediatric cancer survivors, confirming that bedtime use of digital media may be associated with reduced sleep duration [73]. Therefore, regulating access to digital devices may be advisable for PPC patients who routinely use them at night and experience sleep-related adverse effects. At the same time, the use of verified digital tools for relaxation or distraction has recently been approved as an intervention to improve sleep in this population and may be beneficial [74].

#### 4.4.5. Co-Sleeping

Co-sleeping refers to a parent spending the night in the same room as their child and sharing the same sleeping surface, commonly known as bed sharing. Extended co-sleeping can lead to the development of a conditioned response, in which the child becomes dependent on the presence to initiate and sustain sleep [75]. As a result, the child may have trouble falling or staying asleep on his own without their parents present. An investigation focusing on children receiving ongoing chemotherapy for acute myeloid leukemia discovered that parents frequently observed a higher quality of sleep in their children when they co-sleep at home [76]. Preliminary data also highlighted that parents and children requiring PPC often share the same bed [62], suggesting that co-sleeping may be another aspect to consider. While standard sleep hygiene recommendations typically advise limiting co-sleeping, in PPC, such practices are often necessary to ensure adequate care and may provide comfort, reduce anxiety, distract from distressing symptoms, or strengthen family bonds. Guidance should therefore be individualized within a family-centered framework, balancing potential sleep disruption with emotional and clinical priorities.

#### 4.4.6. Other Disruptive Care-Setting Factors

In addition to monitoring factors that may disrupt sleep in the hospital environment, home-based settings should also be considered. However, caution is warranted, as interventions and organizational strategies in both hospital and home settings are informed by each patient’s individual needs, ensuring that adjustments to prevent these contributing factors support the child’s comfort as well as clinical requirements.

### 4.5. Additional Implications for Practice and Directions for Future Research

Exploring sleep has been established as a critical component of providing effective PPC. According to recent expert-based recommendations, the full spectrum of sleep disorders should be considered, given the possibility of concurrent occurrence in the same patient [37]. Sleep is a complex phenomenon with both quantitative (e.g., sleep duration, number of awakenings) and subjective (e.g., “restfulness”, “sleep perception”) components, making it sometimes difficult to define and characterize it objectively, especially in children. To address the additional challenges inherent in PPC, a practical, structured approach that accounts for the child’s medical complexity and the caregiver’s burden seems effective [30].

#### 4.5.1. Assessment Phase

##### Comprehensive Clinical History

A structured, detailed clinical history remains fundamental. Core elements include the child’s sleep routine and patterns, including bedtime and wake time, sleep latency, total sleep duration, frequency and duration of night awakenings, and diurnal naps. Assessment should also consider potential contributors related to comorbidities, including pain, epilepsy, irritability, anxiety, and mood disorders, as well as the type and intensity of nighttime care interventions and functioning of medical devices. Sleep characteristics, along with indirect indicators such as crying, changes in physiological parameters, or signs of discomfort, may provide insight into underlying acute or chronic conditions (e.g., infection, constipation, gastrointestinal reflux) contributing to sleep disruption. In addition, particular attention should be paid to concomitant medication regimens [37,77].

##### Child Self-Reported Sleep Characteristics

Where clinically and developmentally feasible, incorporating child self-report can be valuable, as it provides direct insight into the subjective experience of sleep [78]. Although validated self-report instruments specifically designed for children with LLCs or LTCs are not yet available, general pediatric sleep questionnaires may still provide useful information while awaiting PPC-specific validation.

##### Caregiver-Reported Sleep Characteristics

Caregiver-reported sleep characteristics—captured through structured interviews, sleep diaries, validated questionnaires, or short screening tools—offer essential contextual information regarding behavioral patterns, environmental triggers, and nighttime caregiving workloads [37,78]. In PPC, such subjective reports are not merely supplementary but often represent the primary source of clinically meaningful data, particularly for nonverbal children.

While numerous sleep questionnaires are available for general pediatric populations, relatively few instruments have been developed to evaluate the unique sleep challenges encountered in PPC. This limited availability of specialized tools highlights the need for further development and validation of comprehensive sleep assessment questionnaires that are feasible in clinical practice and capable of capturing the nuanced sleep experiences of children with different LLCs and LTCs. The SNAKE is a 54-item questionnaire specifically designed for children with SPMI [79]. It relies on proxy reporting, with parents or other primary caregivers providing information on the child’s sleep over the preceding four weeks. Out of the given items, a total of 23 items can be categorized into one of the five scales: (i) Disturbances going to sleep, (ii) Disturbances remaining asleep, (iii) Arousal and breathing disorders, (iv) Daytime sleepiness, (v) Daytime behavior disorders. The remaining 31 items encompass various aspects such as sleep conditions, sleep duration, efficiency, and overall sleep quality. The SCAC was developed after reviewing items from the SNAKE and other pediatric sleep questionnaires [80]. It evaluated four dimensions: “falling and staying asleep,” “sleep-associated respiration and arousal,” “daytime sleepiness,” and “sleep-associated movements”—based on caregiver reports. Preliminary validation studies indicate satisfactory psychometric properties and good discrimination between problematic and non-problematic sleep. Although both tools show promise, rigorous validation in pediatric patients receiving palliative care is a priority. This population’s unique symptom burden, communication barriers, variable trajectories, and technology dependence may affect the manifestation, detection, and clinical relevance of sleep disturbances.

##### Selective Use of Polysomnography

Polysomnography is considered the gold standard for characterizing sleep-related breathing disorders, REM behavior disorders, and treatment-resistant sleep disruption, yet its use in PPC must be judicious [37,81,82]. Exacerbations of symptoms, the burden of additional hospitalization for both the patient and their family, and issues related to application and unavailability may further complicate its use. Moreover, the multidimensional medical complexities of PPC patients—including variable symptoms, comorbidities, and ongoing treatments—may compromise the accuracy and interpretability of PSG. Consistent with recent international expert consensus, PSG should be reserved for situations where there is strong clinical suspicion of specific disorders, results are expected to directly alter management, or when particular concerns such as airway safety or nocturnal hypoventilation exist—rather than as a routine assessment [37]. In this population, poor sleep quality may also be partially explained by the presence of sleep-disordered breathing, including both central and obstructive sleep apnea related to neurological impairment, neuromuscular weakness, or craniofacial abnormalities, for which polysomnography may represent a useful diagnostic tool when clinically appropriate.

##### Actigraphy

Actigraphy is a small, typically wrist or ankle-worn sensor that contains an accelerometer capable of detecting and recording movement data in multiple dimensions, which are then translated into digital information to assess sleep–wake patterns, physical activity levels, and circadian rhythms [83]. It is designed to be non-intrusive and comfortable for the wearer, making it a valuable, non-invasive method for monitoring sleep–wake patterns over extended periods in the child’s natural environment.

Recent evidence suggests that actigraphy may be feasible even in children receiving PPC, yielding results comparable to PSG for core sleep parameters such as sleep onset, offset, wake after sleep onset, total sleep time, and sleep efficiency, though it may slightly overestimate total sleep time and efficiency [83]. However, actigraphy may have important limitations in certain clinical conditions: its reliance on movement can misclassify sleep in children with severe motor impairment, limited spontaneous movement, or prolonged nocturnal immobility, and medical devices or nighttime care routines may introduce artifacts. Indeed, Mercante et al. found no agreement on the routine feasibility or reliability of this test in this population [37]. Further research is needed to clarify the accuracy and clinical utility of actigraphy across the heterogeneous PPC population, including those with neuromotor impairments or device-assisted ventilation.

#### 4.5.2. Management of Sleep Disturbances

##### The Importance of a Multidisciplinary Team

Addressing sleep disorders in PPC is inherently challenging due to their multifactorial nature, the marked heterogeneity of the population, the potential refractory course of sleep problems, and the paucity of condition-specific evidence to guide management. As a result, treatment has frequently relied on clinical judgment and experiential knowledge, contributing to uncertainty for both clinicians and families. Effective management requires a multidisciplinary approach that combines tailored non-pharmacological and pharmacological interventions and integrates expertise from PPC clinicians, nurses, psychologists, and sleep medicine specialists, who guide assessment, interpret objective sleep data, and support treatment planning [37].

##### Non-Pharmacological Interventions

In pediatrics, non-pharmacological interventions—such as sleep hygiene education, cognitive-behavioral therapy, relaxation techniques, and behavioral or environmental modifications—have been shown to effectively improve sleep quality and constitute a critical component in the management of sleep disturbances. These interventions have also been recognized as a cornerstone of sleep management in PPC. The 2024 Delphi consensus endorsed structured pre-sleep routines, parent-led behavioral strategies, and daytime physical or cognitive stimulation as effective approaches, with high expert agreement [37]. Specific strategies include establishing consistent bedtime rituals, guided relaxation or mindfulness exercises, age-appropriate cognitive or sensory activities during the day, and caregiver-supported behavioral interventions to reinforce healthy sleep patterns. Implementation should be individualized according to the child’s medical complexity, developmental stage, and caregiver capacity.

##### Sleep Hygiene

Sleep hygiene education involves teaching patients and their parents about good sleep habits, such as maintaining a regular sleep schedule, developing adequate bedtime routines, avoiding screentime and stimulant drinks, and creating a comfortable sleep environment. Some of the most used include: (i) creating a suitable environment for sleep (such as adjusting the temperature and creating a non-stimulating environment); (ii) maintaining a regular and consistent sleep and wake schedule (e.g., not allowing the child to make up for lost sleep by anticipating bedtime or sleeping later); (iii) creating child–parent interactions that are functional for falling asleep and maintaining sleep (e.g., parents should refrain from responding to their children’s disruptive bedtime behaviors).

However, applying standard sleep hygiene principles to children with LLCs or LTCs faces significant challenges. Consistent schedules may be difficult due to fluctuating symptoms and nocturnal care needs. Limiting stimulation can be constrained by medical devices or nighttime monitoring requirements, and discouraging parental response to night awakenings may be inappropriate when driven by pain, discomfort, anxiety, or life-threatening events. High caregiving burden may further limit families’ capacity to implement multiple behavioral recommendations, and rigid routines may conflict with goals such as comfort, closeness, and bonding. Therefore, in this context, sleep hygiene should include prioritizing achievable changes, aligning recommendations with symptom and medication schedules, coordinating nighttime care to minimize disruptions—when possible, and integrating comfort-based, developmentally appropriate parent–child interactions. Future research should develop PPC-specific sleep hygiene models that account for medical complexity, caregiver burden, and family priorities, and evaluate feasibility, acceptability, and clinically meaningful outcomes.

##### Cognitive Behavioral Therapy for Insomnia (CBT-I)

CBT-I is recommended as the first-line treatment option [84]. Its efficacy has been extensively supported by empirical evidence, mainly in the general adult population [85]. Yet, the suitability of CBT-I protocols for children with LLCs or LTCs has not been evaluated. CBT-I principles require substantial adaptation when applied to children who are non-verbal, bedbound, or technology-dependent, as well as to families already experiencing significant caregiving burden. Once again, conventional CBT-I strategies require substantial reinterpretation and adaptation in this setting, as they may be impractical or even contraindicated for medically complex children. The primary goal should be to minimize arousing or discomfort-inducing stimuli while ensuring clinical safety and effective symptom management, rather than focusing solely on sleep. Interventions should prioritize feasibility, co-designed routines, and incremental changes aligned with caregiver capacity.

##### Pharmacological Interventions

There is a general paucity of high-quality data on pharmacological treatments for sleep problems in children, and this evidence gap is even more pronounced in PPC. Clinical practice remains largely empirical, guided by caregiver reports, case series, and medical opinion. Expert consensus emphasized a multifactorial, stepwise approach, beginning with tailored non-pharmacological strategies to address modifiable sleep disruptors and optimize routines, with pharmacotherapy considered where these measures are insufficient or inappropriate for the child’s clinical context and family’s burden. Specific medication recommendations were less harmonized [37].

### 4.6. Melatonin

Melatonin (N-acetyl-5-methoxytryptamine) is a chronobiotic drug that plays a pivotal role in regulating the sleep–wake cycle. In pediatric practice, it is widely used but often prescribed prematurely or administered without prior implementation of first-line behavioral and sleep hygiene interventions. In the PPC population, melatonin was noted as requiring caution, given differences in regulation, manufacturing practices worldwide, and variability in over-the-counter formulations, which may affect its efficacy and safety [37].

### 4.7. Benzodiazepines and Antihistamines

Benzodiazepines and antihistamines are frequently used for their sedative properties to promote sleep, despite the absence of robust evidence supporting their efficacy [86,87]. These agents are not approved for the treatment of insomnia in the pediatric population, and their use is off-label. Moreover, they carry a significant risk of adverse effects, including daytime sedation, paradoxical reactions, and impaired alertness. In PPC, consensus was reached on limiting their use [37].

### 4.8. Other Pharmacological Agents

Dexmedetomidine has recently emerged as a potential treatment option for sleep disturbances in PPC [37,88]. Early evidence indicates promising tolerability; however, long-term safety data remain limited, and its use should be confined to carefully supervised clinical settings.

Similarly, gabapentin may provide benefit for sleep problems in recurrent irritability/agitation in children with disorders of the central nervous system.

Overall, pharmacotherapy in PPC should be individualized, integrated within a comprehensive care plan that prioritizes symptom control, safety, and QoL, and informed by multidisciplinary assessment. Future research should focus on controlled studies to clarify the role, dosing, and safety of sleep-related pharmacological agents in this vulnerable population.

## 5. Conclusions

By prioritizing the assessment, monitoring, and management of sleep disturbances in PPC, healthcare professionals can help optimize sleep and ultimately improve the QoL for children receiving palliative care. Despite their increasing recognition, significant gaps remain. First, there is a need to further develop and validate PPC-specific sleep assessment instruments that capture the complexity of symptoms, communication limitations, and caregiver-mediated observations. Second, prospective cohort studies are required to better characterize the natural history of sleep disruption in PPC, identify risk factors, and determine how sleep interacts with pain, epilepsy, device dependence, and psychological burden over time. Third, evaluating the feasibility and effectiveness of adapted non-pharmacological interventions—such as tailored sleep hygiene programs, caregiver training, and modified behavioral strategies—is essential to inform family-centered and scalable approaches. Finally, real-world pharmacology studies are critically needed to assess dosing, safety, and monitoring requirements for commonly used medications.

## Figures and Tables

**Table 1:** Comparative characteristics of included studies.

Study	Design	Country/Setting	Sample Size	Diagnosis	Measures	Outcomes	Child-Related Factors	Environmental Factors	Findings
Dreier et al., 2018 [30]	Cross-sectional observational (logistic + linear regression)	Germany—children’s hospital, hospice, respite care, home PPC	212 children, mean age 10.4 years	Cerebral palsy (26.3%), global developmental delay (17.1%), rare syndromes (13.7%), metabolic/neurodegenerative (10.2%); 55.8% epilepsy	SNAKE	Sleep quality, sleep duration, sleep efficacy, five sleep problem domains	Pain; nighttime repositioning; epilepsy; medical care	Light; noise; TV/radio; open door	Pain increased risk of poor sleep four-fold (OR 4.13); repositioning OR = 3.08; epilepsy predicted daytime sleepiness
Marinetto et al., 2020 [39]	Cross-sectional descriptive	Italy—regional PPC center	142 children; pediatric PPC population	87% non-oncological (neuromuscular, neurological, metabolic, genetic conditions)	Parent-reported questionnaire (adapted from literature, partial SNAKE-derived items)	Nocturnal awakenings, sleep medication, nighttime procedures, caregiver perception	Pain or crying; need to change position; device-related care	Not quantitatively analyzed	30% had 3–6 awakenings/night; pain and posture changes were main triggers
Harputluoğlu et al., 2025 [38]	Cross-sectional	Turkey—PPC unit	76 dyads, 1 month–18 years; majority of mothers as caregivers	Predominantly neurological (cerebral palsy 52.6%); 84.2% bedridden	CSHQ (child) and PSQI (caregiver)	Child sleep disturbances; caregiver sleep quality	Age, gender, diagnosis, age at diagnosis, night feeding and night care	Not primary variable	98.7% screened positive for sleep disorder; the total CSHQ score was not statistically significantly different on the basis of patient’s age, gender, diagnosis, age at diagnosis, night feeding and night care; worsening child sleep correlated with caregiver poor sleep

## Data Availability

Not applicable.
